# Quality control checkpoints for high throughput DNA methylation measurement using the human MethylationEPICv1 array: application to formalin-fixed paraffin embedded prostate tissue

**DOI:** 10.1186/s13104-025-07221-3

**Published:** 2025-07-09

**Authors:** Robert L. O’Reilly, Fleur Hammet, Neil O’Callaghan, Robert J. MacInnis, Damien Bolton, Graham G. Giles, Pierre-Antoine Dugué, Melissa C. Southey

**Affiliations:** 1 Precision Medicine, School of Clinical Sciences at Monash Health, Monash University, Clayton, VIC 3168 Australia; 2 Department of Clinical Pathology, Melbourne Medical School, The University of Melbourne, Melbourne, VIC 3010 Australia; 3 Cancer Epidemiology Division, Cancer Council Victoria, Melbourne, VIC Australia; 4 Centre for Epidemiology and Biostatistics, Melbourne School of Population and Global Health, University of Melbourne, Melbourne, VIC 3010 Australia; 5 Urology, Austin Health, Heidelberg, VIC 3081 Australia; 6 Victorian Heart Institute, Monash University, Clayton, VIC 3168 Australia

**Keywords:** FFPE tissue, Human methylationepic Beadchip, Bisulfite conversion, Quality control, FFPE tissue-derived DNA

## Abstract

**Objective:**

The performance of FFPE tissue-derived DNA on the MethylationEpicV1.0 array can be unpredictable as the protocol only has two quality checks; checkpoint 1 for DNA quantity and checkpoint 2 for DNA quality assessment. We sought to incorporate a third, previously developed bisulfite conversion quality check prior to processing 255 FFPE tissue derived DNA samples.

**Results:**

FFPE tissue-derived DNAs were prepared for 255 prostate tumour specimens and four controls. Checkpoint 1 assessed all samples to have 500ng DNA available for analysis except for two samples that yielded 483 and 486ng. All DNA samples passed both the quality checkpoint 2 and the bisulfite conversion quality assessment checkpoint 3. Assessment of array performance showed one of the 259 (0.4%) DNA samples had less than 90% of probes detected at *p* = 0.05. Controls and replicate showed reliable reproducibility with correlations of 0.981 and 0.994. High quantity and quality measures of the FFPE tissue-derived DNAs (assessed by checkpoint 1 and 2) were likely responsible for 99.6% of DNAs producing high quality EPIC array data. This report suggests that checkpoint 3 has limited value in a setting where the FFPE tissue derived DNA has high quantity and quality measures.

**Supplementary Information:**

The online version contains supplementary material available at 10.1186/s13104-025-07221-3.

## Introduction

Epigenomic mechanisms, including DNA methylation are important molecular features of human variation and disease susceptibility and progression. Current technology has made genome-wide studies of epigenomes possible for large population-based studies that are fundamental to translating new epigenomic discoveries into improved public health. In the context of most epidemiological and clinical studies of cancer, archival formalin-fixed paraffin-embedded (FFPE) tumour material is an important yet challenging (low quantity and highly degraded) source of DNA for epigenomic profiling [1, 2].

DNA methylation can be measured via a variety of technologies including targeted gene panel sequencing, whole genome bisulfite sequencing and DNA methylation arrays [3, 4], the latter of which has been attractive to studies with large sample sizes due to reduced cost and automation. The Illumina HumanMethylation450 [5] and the updated Human MethylationEPIC arrays (V1 and V2) (with almost twice the CpG targets in comparision to the 450 K), have been used in numerous studies in the past decade. To measure genome-wide DNA methylation in FFPE tissue-derived DNA on the EPIC array, the DNA samples undergo bisulfite conversion at the bench that converts unmethylated cytosines into uracils through deamination without converting methylated cytosines. PCR then converts uracils into thymines resulting in distinguishable differences when compared to reference genomes [6]. This process is critical to the quality of data generated by the arrays as conversion can be incomplete and DNA loss can be considerable as DNA degradation can occur due to the low pH and high temperatures in the process [7]. In addition, FFPE tissue-derived DNA is inherently degraded which can also impact the sensitivity and specificity of the bisulfite conversion method.

Illumina’s standard array processing protocol has two quality assessment steps; the first being DNA quantification and second the Infinium HD FFPE qPCR quality assessment (both preceding the bisulfite conversion). In the Illumina protocol no assessments are performed to determine the success of the bisulfite conversion before progressing onto the array. To address this, Wong et al. [8] developed a quality check that determined whether bisulfite conversion was successful on FFPE breast tumour DNA. The authors showed that excluding the 9% of samples that failed the post bisulfite conversion quality check (checkpoint 3) resulted in a 99% success rate for DNA samples that progressed to the array analysis, saving cost.

Here, we incorporated checkpoint 3 into a protocol to measure the genome-wide methylation of 259 FFPE prostate tumour enriched DNA samples [8, 9].

## Methods

### The lifestyle and genetic risk factors for prostate cancer study

The Lifestyle and Genetic Risk Factors for Prostate Cancer Study (LGRFPCS) recruited Victorian residents through the Victorian Cancer Registry and urology clinics in Victoria. Participants were males aged 20–79 years diagnosed between 2009 and 2014 with aggressive prostate cancer, using the adopted International Consortium for Prostate Cancer Genetics (ICPG) definition [10]. All participants provided informed consent to participate in the LGRFPCS. Diagnostic prostate tissues (*n* = 255) were collected from one Melbourne-based pathology laboratories and stored as 10 μm sections.

### DNA extraction from FFPE prostate tumour material

Haematoxylin and Eosin (H&E) stained sections of prostate tumours were reviewed by a histopathologist to identify and mark up areas of interest. These identified areas were then transcribed onto the back of sequentially cut 10 μm methyl green stained (1%) sections before the areas were macrodissected and placed into microcentrifuge tubes. DNA was extracted from the macrodissected tissue using Qiagen’s QIAamp DNA FFPE kit (Qiagen, Germany) as per the manufacturer’s instructions, except that the tumour material was incubated in Buffer ATL at 56 °C for 48 h, with an additional 20 µl of Proteinase K (20 mg/ml) after 24 h to maximise proteolytic digestion. FFPE tumour-enriched DNA was eluted twice in Buffer ATE to achieve a final elution volume of 20 µl, as described in Wong et al. [8].

### Assay controls

To control for possible processing batch effects, a high molecular weight genomic DNA from a lymphoblastoid cell line (HMW control) was included in each of the three batches. One sample replicate (BeadChip control) derived from FFPE tumour material was added on two bead chips within the same batch to assess variation between bead chips. Thus, there were 255 unique FFPE tumour-derived DNA samples and a total number of 259 DNAs processed on the array.

### QC check point 1: qubit quantification

FFPE tumour-enriched DNA were quantified using a Qubit^®^ dsDNA BR Assay kit on the Qubit^®^ Fluorometer (Life Technologies, CA, USA) as per manufacturer’s protocol. 500ng of FFPE tissue-derived DNA was prepared, whenever possible, to proceed to QC checkpoint 2.

### QC checkpoint 2: infinium HD FFPE qPCR

DNA samples were processed in 3 batches (batches 1 and 2 contained 96 DNAs and batch 3 contained 67 DNA samples) in 96-well plates. The FFPE prostate tumour-derived DNA samples were assessed for quality on a QuantStudio™ 7 Flex Real-Time PCR System (Applied Biosystems, CA, USA) following Illumina’s Infinium HD FFPE qPCR protocol (Illumina, San Diego, CA, USA). As described in Wong et al. [8] samples passed this QC checkpoint if the averaged Ct sample minus the QCT control was within 6 cycles *AND* if the NTC negative control minus the QCT control was greater than 10 cycles:

**Ct**_**Sample**_**– Ct**_**QCT control**_**= ∆Ct ≤ 6 cycles***AND* if **Ct**_**NTC**_**– Ct**_**QCT control**_**= ∆Ct > 10 cycles**.

### Bisulfite conversion and FFPE derived DNA restoration

The prostate FFPE derived tumour-enriched DNA underwent bisulfite conversion and restoration as described previously [8, 11]. Restored bisulfite converted samples had a final elution volume of 10 µl.

### QC checkpoint 3: bisulfite conversion assessment

Wong et al., reported QC checkpoint 3 protocol was used to determine the success of the bisulfite conversion [8]. The qPCR assay in QC checkpoint 3 targets a 134 bp region of the breast cancer 1 (*BRCA1*) gene (GenBank: L78833.1). The primer sequences correspond to converted cytosines (represented by lower-cases) (forward sequence: 5′ tAA GGT AtA ATt AGA GGA TGG GAG GGA t; reverse sequence: 5′ aaC AAA CTC Aaa TAa AAT TCT TCC TC) thereby providing a specific quantifiable assay to determine the degree of converted cytosines present in the test DNA.

The qPCR assay was performed on a QuantStudio™ 7 Flex Real-Time PCR System (Applied Biosystems, CA, USA) with PCR cycling parameters previously described [8]. Successful bisulfite conversion was determined when the difference between the CT_UCcontrol_ by the CT_Sample_ is greater than 4 PCR cycles.

**CtSample– CtUCcontrol = ∆Ct ≥ 4 cycles**.

Samples that passed this QC checkpoint 3 were then processed on the Infinium MethylationEPIC v1.0 beadchip arrays following the manufacturer’s protocols and scanned using Illumina’s ISCAN (Illumina, San Diego, CA, USA).

### Data analysis

Analysis of the qPCR data for the QC checkpoints 2 and 3, as well as the array performance (% CpG detected), was conducted in Excel (Version 16.0.14332) using the protocol published previously [8]. To generate beta values the ENmix (Version 1.38.01) pipeline was used to pre-process and normalise the methylation data, which includes background correction, dye-bias correction, and probe-type bias correlation, and has excellent reported performance [12]. For annotation we used Illuminas’s Infinium Methylation Epic V1.0 B4 GRCh37 manifest file. Analysis of beta values for the control replicates mean variation and correlation was carried out using R (Version 4.3.2) using Pearson’s correlation. For the comparison of more than two replicates, a pairwise matrix was performed and the lower triangle extracted (**Supplementary Script**). The average was then calculated from these values.

## Results

### QC checkpoints

DNA extractions of FFPE tumour tissues enabled 500 ng of input DNA for all samples, except for two that had starting quantities of 483 ng and 486 ng. All 259 samples passed the Infinium HD FFPE QC checkpoint 2. Bisulfite conversion of the 259 samples was conducted in three batches where all samples passed the previously reported [8] QC checkpoint 3 threshold. When analysing the performance of the array by determining the percentage of CpGs probes detected, 258/259 samples passed Illumina’s quality threshold of > 90% of probes detected at *p* = 0.05. One sample had 88% of CpGs detected.

The HMW DNA control across all batches was consistent between replicates (Table 1), with > 97% of CpGs detected at *p* = 0.05. The BeadChip control replicates had consistent % CpGs detected of 98.2% and 98.4%.

**Table 1:** Assessment of array performance, in terms of % of CpGs detected

Sample ID	Batch number	CpG % detected (*p* = 0.05)
HMW DNA control	1	99.9
HMW DNA control	2	99.8
HMW DNA control	3	97.6
Beadchip control	3	98.2
Beadchip control	3	98.4
Sample1 *	3	99.2
Sample2 *	3	98.1
Sample3 ^#^	2	88.0

* the 2/259 samples that had less than 500 ng of input DNA;

# the 1/259 sample that failed the CpG% >90% at *p* = 0.05 threshold on the array performance analysis

### Sample analysis

The two samples, Sample1 and Sample 2, for which the input DNA was below 500 ng (483ng and 486ng respectively) performed well on the EPIC array with 98% of CpG probes detected (Table 1). Out of the ~ 831k CpG sites mapped to the manifest file, these two samples had very few CpG sites with missing probes (59 and 1431 respectively). No obvious relationship was found between quantity (DNA yields (QC1)) and quality ΔCt values (QC2) (Supplementary Fig. 1).

**Fig. 1:**
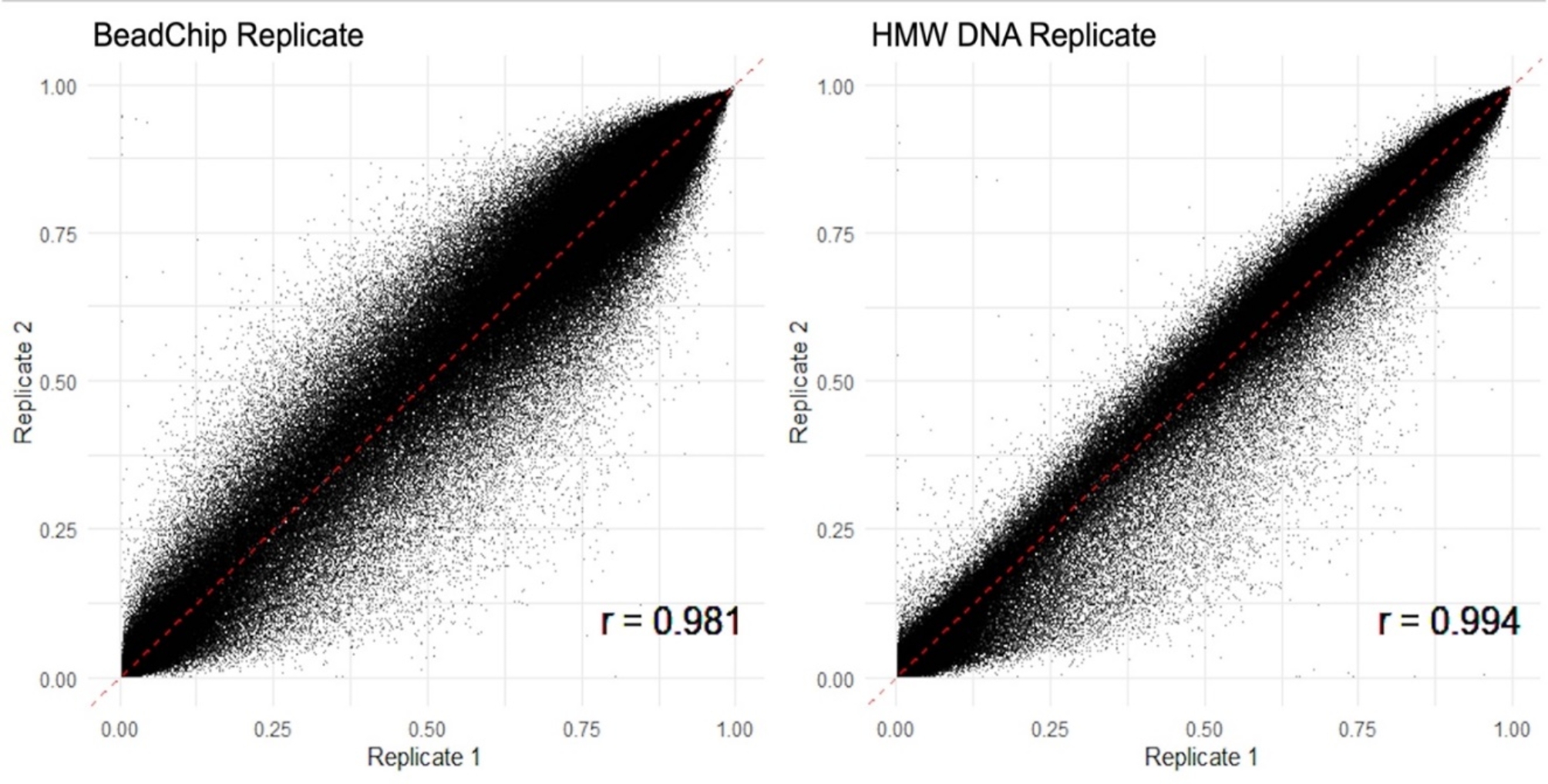
Scatterplot depicting the correlation of beta values between the control replicates assayed

**Fig. 2:**
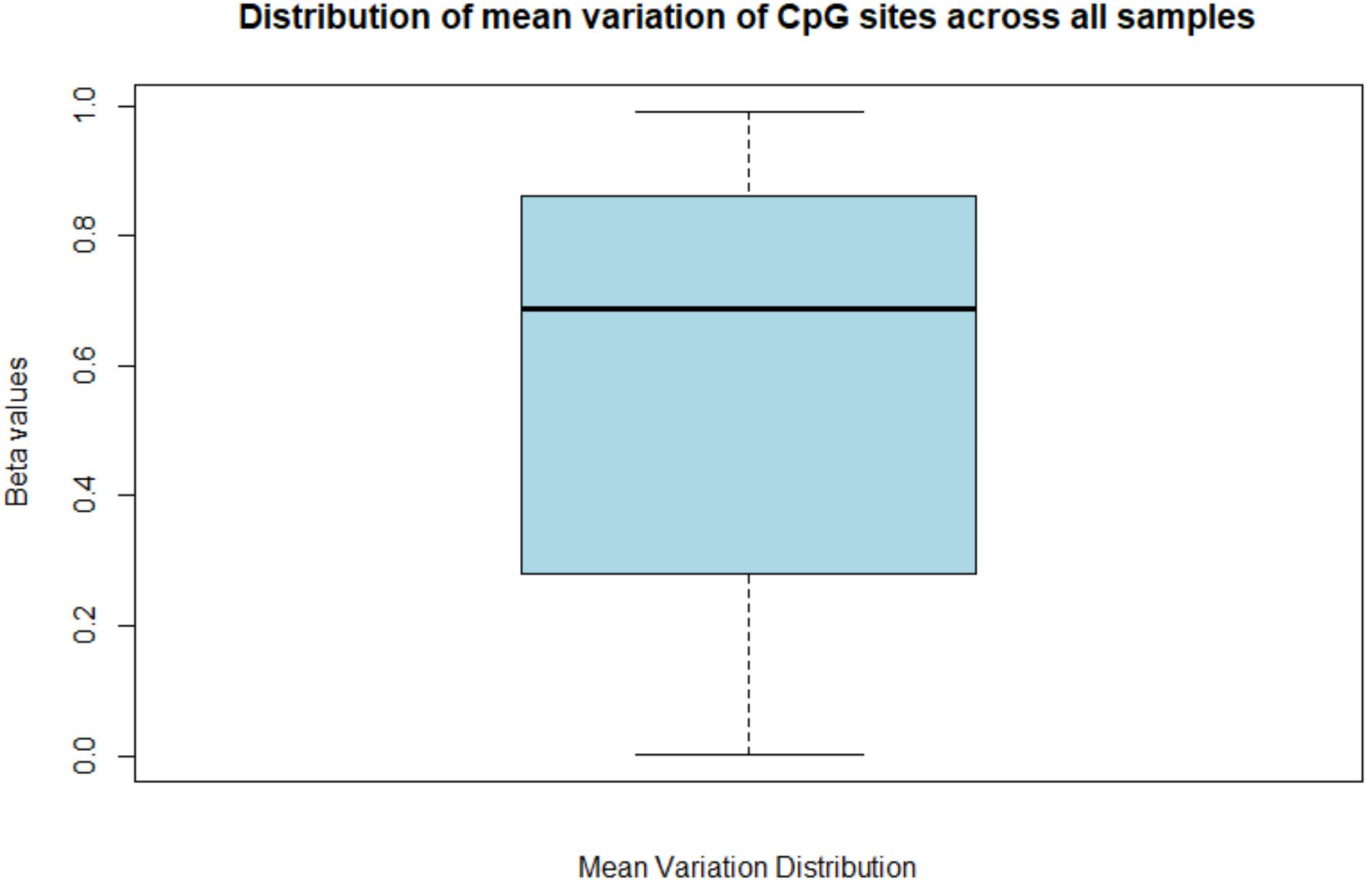
Distribution of mean variation of CpG sites across all samples. Black line shows a median of 0.688

The replicates of the HMW DNA control and the BeadChip control had strong correlations of 0.994 and 0.981, respectively (Fig. 1). The overall correlation between samples was 0.933, with a standard deviation of 0.022. The mean variation between all samples was 0.570, 0.026 between HMW DNA control replicates, and 0.045 between BeadChip control replicates (Supplementary Table 1). Distribution of mean variation of CpG sites across all samples shows slight skewness with a median of 0.688 (Fig. 2).

## Discussion

We demonstrated the incorporation of an additional QC checkpoint for genome-wide DNA methylation profiling using the EPIC array of 255 FFPE tissue-derived tumour DNA samples and four controls (*n* = 259). Overall, none of the three QC checkpoints identified any samples for exclusion and only one sample (0.4%) failed Illumina’s array performance threshold of individual probe detection greater than 90% at *p* = 0.05. The FFPE tissue-derived DNA sample that failed the array performance had passed checkpoint 1–3, indicating that this failure may be a non-systematic error that occurred during array processing. Despite this, the overall success rate for the 259 samples remains high at 99.6%. The controls showed high reproducibility with consistent array performance across batches and had strongly correlated beta values [13, 14] and relatively high variation between samples.

This report shows the successful application of the bisulfite conversion assessment (checkpoint 3) for prostate tumour FFPE material, initially developed by Wong et al. [8] for breast tumour FFPE material. Checkpoint 3 was easily incorporated to enable informed decisions on whether each DNA sample is suitable for the expensive downstream EPIC array processing. However, in contrast to the report of Wong et al., all of the prepared FFPE tissue-derived DNA samples extracted and processed in this study resulted in high quantity (checkpoint 1) and quality (checkpoint 2); were robust to the bisulfite conversion (checkpoint 3); and all but one produced high quality EPIC array data.

This report suggests that the value of checkpoint 3 is likely limited to FFPE tissue-derived DNA resources that have highly variable quantity and quality measures. Of the 474 FFPE tissue derived DNAs included in Wong et al., five did not generate any detectable DNA at checkpoint 1, and 42 (9%) did not pass the bisulfite-specific qPCR checkpoint 3 [8]. The FFPE breast tumour tissues in Wong et al., were collected from many diagnostic laboratories and thus variation in fixation, processing, and storage protocols are highly likely. Tissue storage time also ranged from 8 to 20 years. The FFPE prostate tumour material included in this study were all collected from one Melbourne-based pathology laboratory with storage time ranging from 8 to 13 years which, in part, is likely to explain their consistently high performance in this assay.

### Limitations

A limitation of the study is the limited number of replicate samples across batches. Evaluation of reproducibility would be enhanced if more replicates were available for analysis, e.g. a larger number of sample replicates between plates, and more than one BeadChip replicate in 33. However, the relative high cost per sample limits the number of possible replicates. The strong correlation between replicates in this study suggests a reliable result without needing further replicate samples. Two samples had lower input DNA (483 and 486ng) compared to all other samples (500 ng). These two samples were from sections that had small foci of tumours for which it was not possible to extract more DNA. As the two samples were well above the manufacturer’s minimum input DNA for this array (250 ng), they were included in the study.

## Electronic supplementary material

Below is the link to the electronic supplementary material.

Supplementary Material 1

Supplementary Material 2

## Data Availability

Code and Beta values used to calculate correlation between controls (BeadChip and HMW control) are provided in the supplementary data file. Expressions of Interest from researchers to use the resources of the Lifestyle and Genetic Risk Factors for Prostate Cancer Study (LGRFPCS) can be submitted to PEDIGREE. PEDIGREE Project - Cancer Council Victoria (cancervic.org.au).
