# Supplementary material for: Quality control checkpoints for high throughput DNA methylation measurement using the human MethylationEPICv1 array: application to formalin-fixed paraffin embedded prostate tissue
Source: BMC Res Notes. 2025 Jul 9;18:280. doi: 10.1186/s13104-025-07221-3 (PMC12239449; doi:10.1186/s13104-025-07221-3)
Supplement: Supplementary file 2 — Supplementary Material 2 [file 13104_2025_7221_MOESM2_ESM.docx]

### Supplementary Material

**Supplementary Table 1:** Mean variation of beta values between replicates across all ~831k CpG sites. N indicates the number of samples used for that replicate type throughout the process.

| **Replicate** | **N** | **Mean Variation** |
| --- | --- | --- |
| Cell-line control | 3 | 0.026 |
| BeadChip control | 2 | 0.045 |
| Overall variation (all samples) | 258* | 0.570 |

*After removing one sample that did not pass the array performance

**
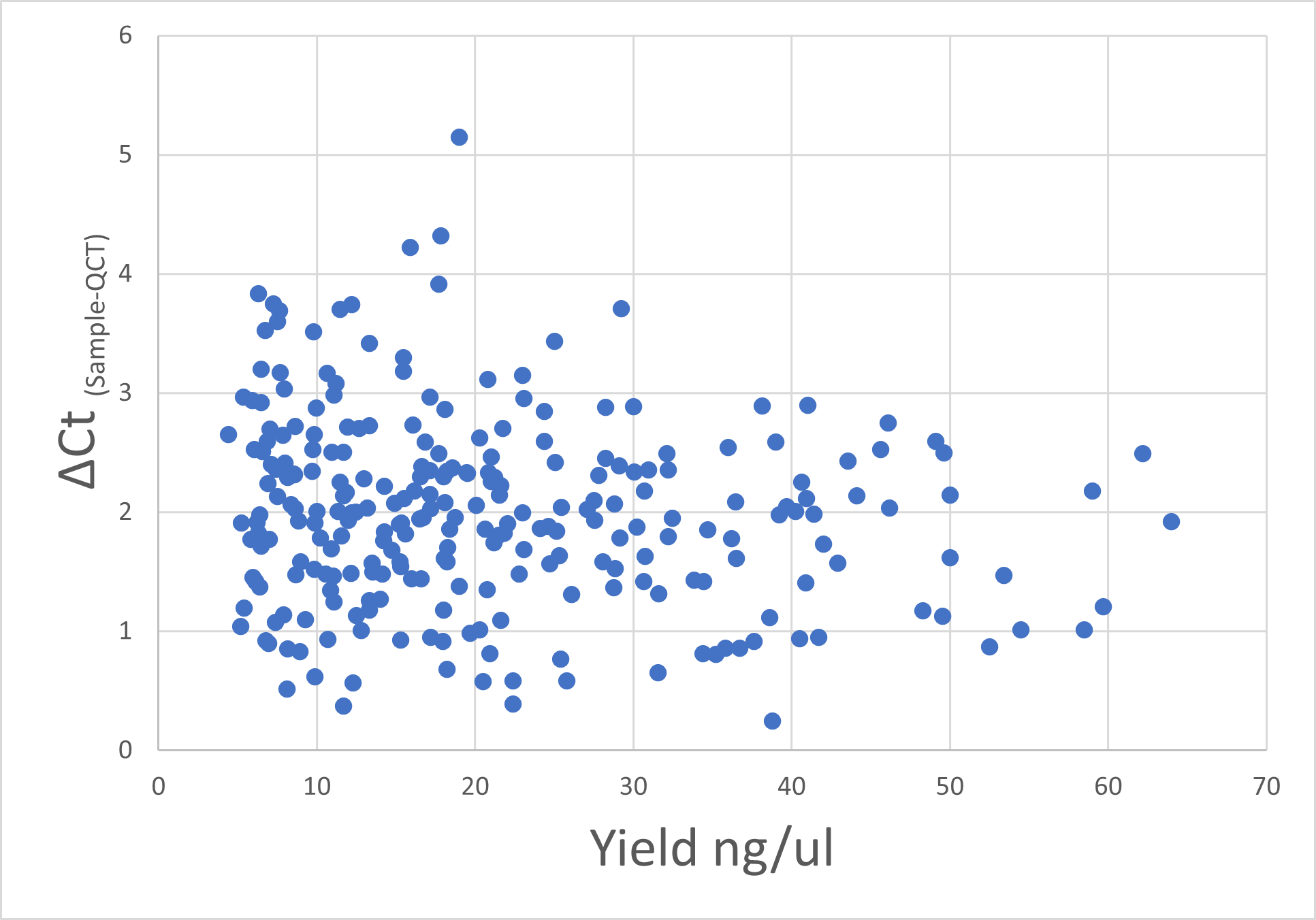
**

**Supplementary Figure 1: A comparison between QC1 DNA yields (ng/ul) and QC2** **ΔCt values as representation for quantity vs quality.**

**Supplementary Script:**

#R code used for calculation used on control's beta values used in the manuscript

#"Quality control checkpoints for high throughput DNA methylation measurement using the

#Human MethylationEPICv1 array: application to formalin-fixed paraffin embedded prostate tissue".

#11.06.2024

#Robert O’Reilly and Pierre-Antoine Dugue

#loading packages

library(pacman)

pacman::p_load(TCGAbiolinks,ENmix,minfiData,IlluminaHumanMethylationEPICmanifest, IlluminaHumanMethylationEPICanno.ilm10b4.hg19,TxDb.Hsapiens.UCSC.hg19.knownGene, org.Hs.eg.db,devtools, M3C, dplyr, ggplot2, scattermore, gridExtra, cowplot, tidyverse)

#Reading in the idat files

path <- file.path('path/to/idat files')

rgSet <- readidat(path = path, recursive = TRUE, force = TRUE)

QCinfo <- QCinfo(rgSet)

QCinfo$badsample

QCinfo$outlier_sample

# None

detP <- QCinfo$detP;

beta_1=mpreprocess(rgSet,nCores=6,qc=TRUE,fqcfilter=TRUE, rmcr=TRUE,impute=TRUE)

# loading sample sheet for sample IDs

FulldataIndexNames <- read.csv("samplesheet.csv")

REQ645fulldf <- beta_1

#adding TAPC names to the array id

CompletedFullSamplelistREQ645 <- REQ645fulldf %>%

left_join(FulldataIndexNames, by = c("ID" ="array_id"))

##Annotation

#adding the manifest csv to annotate the CpG sites

manifest <- read.csv("MethylationEPIC_v-1-0_B4-GRCh37-manifest-file.csv", stringsAsFactors = FALSE)

matched_sites <- merge(manifest, CompletedFullSamplelistREQ645, by.x = "IlmnID", by.y = "TAPC")

annotations <-matched_sites[, c("IlmnID","CHR","UCSC_RefGene_Name")]

CompletedFullSamplelistREQ645_annotated <- merge(CompletedFullSamplelistREQ645, annotations, by.x = "CpG_sites", by.y = "IlmnID", all.x = TRUE)

##subsetting controls and renaming

Controls_dataset <- subset(CompletedFullSamplelistREQ645_annotated,select = c("CpG_sites", "CHR", "UCSC_RefGene_Name", "206949980014_R05C01", "206949980045_R08C01","206949970001_R01C01","206949980045_R02C01", "206949980083_R08C01" ))

colnames(Controls_dataset)[colnames(Controls_dataset) %in% c("206949980014_R05C01", "206949980045_R08C01", "206949970001_R01C01")] <- c('Cell_line_control1', 'Cell_line_control2', 'Cell_line_control3')

colnames(Controls_dataset)[which(colnames(Controls_dataset) %in% c('BeadChip_control1', 'BeadChip_control2'))] <- c('206949980045_R02C01', '206949980083_R08C01')

#Create correlation matrix between 3 cell line controls. This has to be matrix because comparing more than 2 columns

cor_matrix <- cor(Controls_dataset[, c(4, 5, 6)], use = "complete.obs", method = "pearson")

mean_cell_line_correlation <- mean(cor_matrix[lower.tri(cor_matrix)], na.rm = TRUE)

mean_cell_line_correlation <- round(mean_cell_line_correlation, 3)

> mean_cell_line_correlation

[1] 0.994

#Calculate correlation between the two BeadChip control replicates

BeadChip_correlation <- cor(Controls_dataset[[7]], Controls_dataset[[8]], use = "complete.obs", method = "pearson")

BeadChip_correlation <- round(BeadChip_correlation, 3)

> BeadChip_correlation

[1] 0.981

#Calculating mean variation between Cell-line Controls. Again, needed a slightly

#different method for the 3 replicates compared to the BeadChip

CelllineTotalMeanVari <- Controls_dataset %>%

rowwise() %>%

mutate(

variation = mean(

c(abs(c_across(c(4, 5))[[1]] - c_across(c(4, 5))[[2]]),

abs(c_across(c(4, 6))[[1]] - c_across(c(4, 6))[[2]]),

abs(c_across(c(5, 6))[[1]] - c_across(c(5, 6))[[2]])),

na.rm = TRUE

)

) %>%

ungroup()

Cellline_variation <- mean(CelllineTotalMeanVari$variation, na.rm = TRUE)

Cellline_variation <- round(Cellline_variation, 3)

> Cellline_variation

[1] 0.026

#Calculating mean variation between BeadChip Controls

BeadChip_variation <- Controls_dataset %>%

mutate(variation = abs( `BeadChip_control1` - `BeadChip_control2`))

BeadChip_variation <- mean(BeadChip_variation$variation, na.rm = TRUE)

BeadChip_variation <- round(BeadChip_variation, 3)

> BeadChip_variation

[1] 0.045

#scatterplot for cell-line control

Cellline_scatterplot <- ggplot(Controls_dataset, aes(x = Controls_dataset[[4]], y = Controls_dataset[[5]])) +

geom_scattermore(alpha = 0.5, size = 1) +

geom_abline(intercept = 0, slope = 1, color = "red", linetype = "dashed") +

labs(title = "Cell-line Replicate ",

x = "Replicate 1",

y = "Replicate 2") +

theme_minimal() +

coord_cartesian(xlim = c(min(Controls_dataset[[4]]) * 2.0, max(Controls_dataset[[4]]) * 2.0),

ylim = c(min(Controls_dataset[[5]]) * 2.0, max(Controls_dataset[[5]]) * 2.0)) +

geom_text(aes(x = 0.9, y = 0.10, label = paste("r =", round(mean_cell_line_correlation, 3))), color = "black", size = 6)

#scatterplot for the beadchip control

BeadChipscatterplot <- ggplot(Controls_dataset, aes(x = Controls_dataset[[7]], y = Controls_dataset[[8]])) +

geom_scattermore(alpha = 0.5, size = 1) +

geom_abline(intercept = 0, slope = 1, color = "red", linetype = "dashed") +

labs(title = "BeadChip Replicate ",

x = "Replicate 1",

y = "Replicate 2") +

theme_minimal() +

coord_cartesian(xlim = c(min(Controls_dataset[[7]]) * 2.0, max(Controls_dataset[[7]]) * 2.0),

ylim = c(min(Controls_dataset[[8]]) * 2.0, max(Controls_dataset[[8]]) * 2.0)) +

geom_text(aes(x = 0.9, y = 0.10, label = paste("r =", round(BeadChip_correlation, 3))), color = "black", size = 6)

#Plots both the beadchip control and Cell-line replicates next to each other.

Controls_scatterplots <- grid.arrange(BeadChipscatterplot,Cellline_scatterplot, ncol = 2)
